# Arterial spin labeling MRI in assessing cerebral blood flow changes due to hypertension: a systematic review

**DOI:** 10.1097/HJH.0000000000004150

**Published:** 2025-09-26

**Authors:** Sathya Sabina Muthu, Suresh Sukumar, Rajagopal Kadavigere, Shivashankar K.N., K. Vaishali, Ramesh Babu M.G., Hari Prakash Palaniswamy, Abhimanyu Pradhan, Winniecia Dkhar, Nitika C. Panakkal, Sneha Ravichandran, Dilip Shettigar, Poovitha Shruthi Paramashiva

**Affiliations:** a Department of Medical Imaging Technology, Manipal College of Health Professions; b Department of Radiodiagnosis and Imaging, Kasturba Medical College; c Department of General Medicine, Kasturba Medical College; d Department of Physiotherapy, Manipal College of Health Professions; e Department of Department of Basic Medical Sciences, MMMC; f Department of Speech and Hearing, Manipal College of Health Professions; g Division of Yoga, Centre for Integrative Medicine and Research, Manipal Academy of Higher Education, Manipal, Karnataka, India

**Keywords:** arterial spin labeling MRI, blood pressure, brain, brain perfusion, cerebral blood flow, cerebral perfusion, high blood pressure, hypertension, neurovascular disease

## Abstract

Hypertension is a significant risk factor for cerebrovascular diseases, affecting cerebral blood flow (CBF) and brain health. Reduced CBF in hypertensive individuals is linked to cognitive decline and neurodegenerative diseases. Arterial spin labeling (ASL) MRI offers a noninvasive method to assess these changes. This systematic review consolidates evidence on the impact of hypertension on CBF using ASL-MRI. A comprehensive search across PubMed, Scopus, Embase, and Web of Science, following PRISMA 2020 guidelines, included studies on adults with hypertension reporting CBF measurements. Findings indicate that hypertension reduces CBF in various brain regions, with improvements seen after antihypertensive treatment. ASL-MRI may be a valuable tool for monitoring treatment effectiveness and brain health. However, most studies were conducted in high-income countries and elderly populations, emphasizing the need for further research in younger and low-income settings. Early CBF assessment using ASL-MRI could aid in timely interventions.

## INTRODUCTION

High blood pressure, or hypertension, is a global health condition that affects an individual's health and overall well being and is also a cause of morbidity and mortality worldwide [1]. Persistent blood pressure reading of 140/90 mmHg or higher is generally considered hypertension [2]. Hypertension is a significant risk factor for cardiovascular diseases (CVD) and cerebrovascular diseases, which contribute significantly to premature death [3,4]. The prevalence of high blood pressure variations across different regions and country income groups has been demonstrated by the WHO. As per the WHO, there has been a drastic rise in the number of adults with hypertension from 594 million in 1975 to 13 billion in 2015, with the increase mainly noted in low and middle-income countries [5,6].

Blood pressure levels rise progressively with age due to the cumulative exposure to environmental factors that lead to elevated blood pressure levels over time [7]. The environmental factors contributing to high blood pressure include increased intake of sodium, insufficient potassium in the diet, overweight and obesity, alcohol consumption, and physical inactivity [8]. In addition, environmental factors, genetic predisposition, and adverse conditions in the intrauterine environment are also associated with high blood pressure [9].

Hypertension acts as a silent killer over time that often remains asymptomatic until serious complications and symptoms appear [10]. Persistent high blood pressure over time can affect the vasculature and cause organ damage and disability [11,12]. The organs majorly damaged due to hypertension include the brain, kidneys, eyes, arteries, and heart valves [13,14]. The brain is a vital organ in the body that governs overall well being. The consequences of hypertension on the brain include stroke, small vessel cerebral ischemia disease, vascular dementia, and cognitive impairment [15]. High blood pressure causes arterial wall stress, reduced cerebral blood flow, and disruption of the blood-brain barrier, gradually leading to brain organ damage [16]. Over time, hypertension disrupts the brain's ability to regulate CBF through impaired autoregulation and vascular remodelling, where small cerebral arteries and arterioles thicken and narrow in response to prolonged high blood pressure. This reduces vessel compliance, increases vascular resistance, and limits the brain's ability to adapt blood flow to metabolic demands. Furthermore, hypertension contributes to small vessel disease, which impairs microcirculation and leads to chronic ischemia [17,18].

Adequate blood supply to the brain ensures optimal functioning, but impaired autoregulation and reduced CBF that occurs due to hypertension are significant cerebrovascular risk factors [19]. The CBF can be measured noninvasively using the advanced Arterial Spin Labeling (ASL) MRI. ASL works by magnetically tagging the protons in the blood before they reach the tissue that needs to be imaged [20]. The magnetic labeling of arterial protons is carried out upstream from the volume of interest, at the neck vessels, by applying specific radiofrequency pulses, and images are obtained using rapid image acquisition techniques such as echo-planar imaging (EPI) or 3D ultra-fast sequences [21,22]. The major advantage of ASL-MRI is that it does not require a contrast agent, making it a completely noninvasive technique for quantifying CBF, in contrast to conventional contrast-enhanced perfusion MRI. The CBF is a physiological parameter that determines the delivery rate of oxygen and nutrients to the capillary bed. It is expressed as the volume of blood per volume of tissue per minute (ml 100 g^−1^ min^−1^).

Several studies have explored the impact of hypertension on CBF, yet the findings remain inconsistent, and a comprehensive synthesis of evidence is lacking. Although several reviews have discussed cerebral perfusion imaging and the general applications of ASL-MRI [21–23], to our knowledge, no systematic review has specifically focused on the role of ASL-MRI in assessing CBF alterations associated with hypertension. This review addresses that gap by focusing exclusively on studies employing ASL-MRI to investigate perfusion changes in individuals with hypertension. We aim to consolidate the emerging evidence regarding the effects of hypertension on CBF using ASL-MRI to advance our understanding of CBF alterations in hypertensive individuals and identify specific cerebral regions at risk as a guide for future research on hypertension-related CBF changes.

## METHOD

### Reporting methods and registration

This systematic review follows the Preferred Reporting Items for Systematic Reviews and Meta-Analysis (PRISMA) 2020 statements and guidelines [24]. The review protocol is registered in the International Prospective Register of Systematic Reviews (CRD42024585373).

### Data sources and search strategy

**FIGURE 1:**
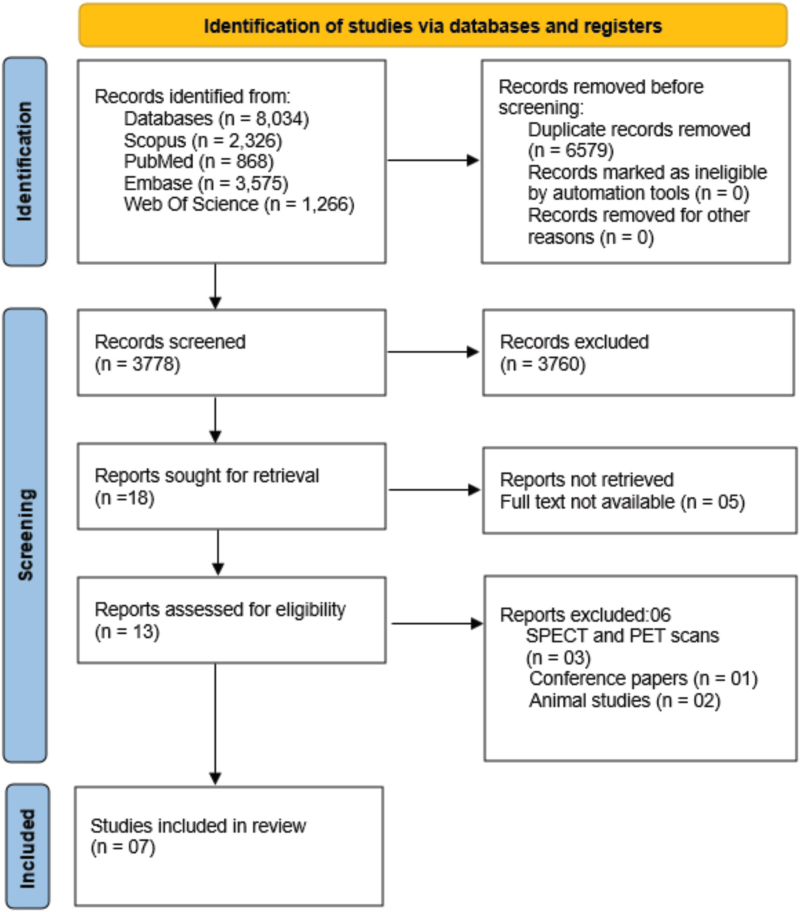
PRISMA flow chart. This flowchart outlines the study selection process, including the number of articles screened, excluded (with reasons), and included in the final analysis.

The computerized English literature search was conducted using electronic databases such as PubMed (Medline), Scopus, Embase, and Web of Science. The search aimed to find data related to cerebral blood flow (CBF) assessment due to hypertension using arterial spin labelling (ASL) MRI. To find the relevant data, the following search strategy was used with Boolean operators: (“High Blood Pressure” [Mesh] OR “High BP” OR Hypertension) AND (“Cerebral Blood Flow” OR “Arterial Spin Labelling MRI”) AND (“Cerebral Perfusion” OR “Brain Perfusion”). Rayyan software screened the articles and reference lists for relevant publications. One author (SSM) independently searched to avoid selection bias. The flow of the article selection process is shown in the PRISMA 2020 flow diagram (Fig. 1).

### Eligibility criteria

The search and extraction of studies were guided by the Population, Exposure, Comparison, and Outcome (PECO) criteria, which served as the benchmark for determining study eligibility [25]. The following shows the PECO criteria and explanation.

1. Population – adults aged 18 years and older diagnosed with Hypertension.
2. Exposure – hypertension, characterized by sustained high blood pressure, typically defined as systolic BP≥140 mmHg or diastolic BP≥90 mmHg.
3. Comparison – nonhypertension, defined as having blood pressure readings below 140/90 mmHg.
4. Outcome – cerebral blood flow (CBF) assessment using ASL-MRI.

### Study selection and data extraction

Two authors (S.S.M. and S.S.) independently screened the titles and abstracts of the citations using the “Rayyan” web application for systematic reviews after removing duplicates. This ensured reliability in study selection and reduced the risk of bias. The level of agreement between the authors was determined based on the number of consistent decisions, and any discrepancies identified were resolved through discussion. If the title and abstract of an article did not provide enough information to assess its relevance, such as lacking details on the study design, population focus, ASL-MRI technique, or outcome measures, the full text was retrieved for further evaluation to assess eligibility. The inclusion criteria focused mainly on studies assessing CBF in hypertensive patients using the ASL-MRI technique. Studies were excluded if they did not focus on CBF as the primary outcome or utilized techniques other than ASL-MRI. If the authors found the study eligible, the data were extracted. The two authors performed data extraction independently, and any discrepancies were resolved through discussion. A custom data extraction sheet was used to extract the following data from the included studies: the study authors and date; the participant characteristics (age, gender); study objectives; and main findings (CBF in different regions of the brain).

### Quality assessment

The quality of the study was assessed using the Joanna Briggs Institute (JBI) critical appraisal tool [26]. The JBI critical appraisal tool was selected because it is a widely recognized and reliable tool for evaluating study quality; it provides a standardized approach to assess methodological rigour, helping to ensure the inclusion of high-quality evidence in systematic reviews [27]. The JBI tool evaluates bias in participant selection, measurement validity, confounding factors, and outcome assessment [26]. A study that answers ‘yes’ to most of the JBI checklist is considered a high-quality study.

### Statistical analysis

Meta-analysis was conducted separately for hypertensive and nonhypertensive groups to evaluate differences in CBF within the frontal lobe and hippocampus. Each study's mean and standard deviation (SD) were extracted, and pooled effect estimates were calculated. A random-effects model was applied to the hypertensive group analysis to account for substantial between-study heterogeneity, as indicated by high *I*^2^ values (>90%). In contrast, a fixed-effects model was used for the nonhypertensive group analysis of the frontal lobe, as no significant heterogeneity was observed (*I*^2^ = 0%). The weighted mean difference and corresponding 95% confidence intervals (CI) were calculated for each analysis. Heterogeneity was assessed using the *Q* statistic and *I*^2^ index. Meta-analysis was not conducted for the hippocampus in the nonhypertensive group or other brain regions due to insufficient data. All analysis were performed using the Jamovi statistical software (version 2.3), with a *P*-value <0.05 considered statistically significant.

## RESULTS

### Search results

A thorough search of articles through four peer-reviewed databases yielded 8034 articles in the initial search. The PRISMA diagram shows the screening of the articles based on the eligibility criteria (Fig. 1). The duplicates were eliminated by screening through the “Rayyan” software for systematic reviews. After duplication removal, 3778 articles were still included for screening, and 18 articles were sought for retrieval and full-text analysis. Seven articles were found eligible based on the inclusion criteria and included in this systematic review. Eleven articles were excluded with appropriate explanations (Fig. 1).

### Study characteristics

All the studies in this systematic review are from different countries, ensuring geographical diversity. All the studies are from high-income and upper-middle-income countries, such as the USA, UK, China, and Russia. None of the studies are from low-income or lower-middle-income countries. Table 1. shows the detailed characteristics of the seven studies included in this review, and their publication dates range from 2008 to 2023. All the studies employed an observational design, with the majority being cross-sectional studies.

**TABLE 1:** Characteristics of the studies included in the review

Author, year	Country	Study design	Study objective	Participants	Methods used for BP assessment	ASL-MRI technique	Outcome measure	Key findings	Limitations
Glodzik *et al.* 2019 [19]	New York	Cross-sectional study	To analyse the relationship between blood pressure and cerebral perfusion.	*N* = 445 Age = Above 50 years Male-38.4% Female-68%	Blood pressure (BP) was measured in a sitting position after the subjects had rested for 5 min using a manual sphygmomanometer in the left upper arm.	The ASL images acquired on the 3T Siemens system and images were subjected to batch processing to generate CBF values from the defined regions of interest (ROIs). This ensured that all voxels with CBF greater than 150 ml/(100g min) were excluded to avoid bias from large blood vessels. ASL parameters-TR-2250ms, TE-2.7ms, slice thickness-1.0 mm, Inversion time-900ms.	Cortical and hippocampal CBF	There was decreased cortical (β= − 0.13, *P* = 0.005 (*B* = −0.047, 95% CI −0.080, −0.015), and hippocampus (*β* = −0.12, *P* = 0.01 (*B* = −0.069, 95% CI −0.121, −0.016) cerebral blood flow with increased systolic blood pressure. Also, the optimal mid-range systolic blood pressure was observed to maximise brain perfusion.	Higher variability in hippocampal CBF measurements was observed, which complicates the analysis.
Alosco *et al.* 2014 [28]	USA	Observational cross-sectional study	To examine the associations among hypertension, cerebral blood flow, and cortical thickness in older adults.	*N* = 58 Age = Above 50 years Female-47%	Diagnostic history of hypertension was ascertained through self-report and, when available, confirmed by medical record review.	ASL scans were Acquired on 3T Siemens system using a proximal inversion with control for off-resonance effect, and perfusion maps were created using MATLAB software. ASL parameters-TR-2500ms, TE-16ms, slice thickness-6 mm, Inversion time-1800ms.	Total Brain perfusion, occipital lobe perfusion, temporal lobe perfusion, frontal and parietal lobe perfusion	Compared to those without hypertension, participants with hypertension exhibited reduced cerebral perfusion in the total brain(47.33 ± 7.14 vs. 49.96 ± 6.57ml/100 g/min), temporal(45.42 ± 8.03 vs. 48.00 ± 6.57ml/100 g/min), occipital lobes(49.99 ± 10.50 vs. 54.21 ± 10.01 ml/100 g/min), parietal lobe(48.27 ± 7.27 vs. 50.83 ± 7.34 ml/100 g/min), and frontal lobe(45.65 ± 7.62 vs. 46.80 ± 6.57 ml/100 g/min). Decreased total and regional cortical thickness was also noted and reduced perfusion predicted decreased cortical thickness in various brain regions.	The study had a relatively modest sample size, limiting the findings’ external validity.
Dai *et al.* 2008 [30]	Pittsburgh	Observational Cross-sectional study	To examine regional cerebral blood flow (rCBF) in normal cognitive-performing subjects with hypertension (HTN) using continuous arterial spin-labelled MRI.	*N* = 41 Mean age = 70–90 years Male-14 Female-27	Individuals with systolic blood pressure >140 or diastolic >90 mm Hg in 2 separate measurements was considered hypertension.	ASL scans were performed using a 1.5T GE Signa system. Continuous arterial spin-labelled MRI was used to measure regional cerebral blood flow. ASL parameters- FoV- 20 cm, TR-700ms, TE-21ms, matrix-64 × 64, slice thickness-5 mm, Inversion time-alternating single and double adiabatic inversions (3.7-s pulse train at 92% duty cycle) was applied.	Regional cerebral blood flow (rCBF) in the subcortical, limbic and paralimbic structures	Normal subjects with hypertension showed decreased rCBF in the putamen (23.1 ± 7.3 vs. 42.6 ± 12.6ml/100 g/min), globus pallidus (25.0 ± 7.0 vs. 44.0 ± 9.66ml/100 g/min), bilaterally, and in the left hippocampus (30.0 ± 11.9 vs. 51.8 ± 12.066ml/100 g/min). In addition, decreased rCBF was observed in the right and left anterior cingulate gyrus, left posterior cingulate gyrus and medial precuneus, left lateral inferior and superior frontal, and inferior parietal, left orbitofrontal, and left superior temporal cortices with *P* < 0.001. These brain regions affected by hypertension are also known to be affected by Alzheimer's disease, suggesting that hypertension may increase the vulnerability of these individuals to develop Alzheimer's disease.	There was no examination of the duration of hypertension or treatment effects on rCBF, which poses a limitation to understanding the long-term impacts of hypertension.
Li *et al.* 2023 [29]	China	Observational study	To explore the association of blood pressure (BP) measurements with cerebral blood flow (CBF) and brain structure in the general population.	*N* = 902 Mean age = 45–60 years Male-53%	The diastolic and systolic BP were measured in triplicate after Participants were seated in a quiet room for five minutes, and the BP measurements were taken at 1-min intervals, and the average value of the Three measurements were used for analysis.	ASL scan was performed on a 3T GE system using a 3D pseudo-continuous ASL sequence, and CBF maps were generated using the 3D ASL Functool software. ASL parameters- FoV- 256 mm, TR-5313ms, TE-10.7ms, slice thickness-4 mm, Inversion time-2525ms, postlabel delay (PLD)-2525 ms	Total and regional CBF and tissue volume	Elevated blood pressure, specifically diastolic blood pressure (DBP), was associated with decreased CBF in the total brain: β = –0.62 (95% CI: –1.14, –0.10), Total grey matter: *β* = –0.71 (95% CI: –1.27, –0.14), Frontal lobe: *β* = –0.72 (95% CI: –1.31, –0.13), Parietal lobe: *β* = –0.92 (95% CI: –1.54, –0.30), Temporal lobe: *β* = –0.63 (95% CI: –1.18, –0.08), Occipital lobe: *β* = –0.69 (95% CI: –1.37, –0.01), and Hippocampus: *β* = –0.59 (95% CI: –1.13, –0.05).	The sample was drawn from a specific community (Kailuan), which may not represent other populations, potentially affecting the external validity of the results.
Christie *et al.* 2022 [32]	UK	Observational cross-sectional study	The study's main objective was to evaluate the association between systemic arterial blood pressure and cerebral perfusion using ASL MRI.	*N* = 740 Age = Above 40 years Male-413 Female-327	Office blood pressure measurements were obtained on the same day When cerebral perfusion was assessed from ASL images.	Cerebral perfusion measurements were performed using ASL-MRI and quantified using a single-compartment model without partial volume correction. ASL parameters- Not specified.	Grey matter and White matter cerebral perfusion	High systemic arterial blood pressure is associated with reduced cerebral perfusion in grey (*P* = 0.01, *R*^2^ = 0.05) and white matter (*P* = 0.001, *R*^2^ = 0.06). In addition, Patients with uncontrolled hypertension on medication had the lowest CBF values in grey matter(39 ± 7 vs. 36 ± 6 ml/100 g/min) and white matter(14.5 ± 4 vs. 13 ± 3ml/100 g/min) compared to participants with normal BP.	Participants were from the UK's largest tri-ethnic study, which may limit the generalizability of the findings to other populations or ethnic groups.
Tryambake *et al.* 2013 [31]	UK	Observational study	To determine the effect of intensive BP lowering on CBF in hypertensive older subjects.	*N* = 37 Mean age = 70–80 years Female-22 Male-15	24-h ambulatory BP were performed At baseline and after 12 weeks of treatment. CBF was measured using a flow-sensitive alternating inversion Recovery (IR) ASL sequence.	The ASL scan was performed on the 3T Philips medical system, and CBF was measured using a flow-sensitive alternating inversion Recovery (IR) ASL sequence. ASL parameters-TR-4s, TE-26 ms, slice thickness-6 mm, Inversion time-1700ms,	Grey matter CBF	Grey matter CBF increased significantly in the intensive group compared to the usual target in older adults (7 ± 11 vs. −3 ± 9ml/100 g/min); thus, Effective blood pressure treatment reduces stroke and myocardial infarction risks.	The study focused on participants over 70 years old, potentially excluding valuable insights into the association between blood pressure and brain perfusion in younger individuals. Baseline systolic BP was slightly higher in the usual care group. Also, the CBF response to orthostatic challenge was not studied.
T.M *et al.* 2018 [33]	Russia	Observational study	To assess cerebral perfusion in middle-aged untreated patients with hypertension	*N* = 73 Mean age = 40–59 years Male-28 Female-45	24-h Ambulatory blood pressure (BP) monitoring was performed.	ASL scan performed on 3T Siemens system using pulsed Arterial Spin Labeling (PASL) technique to obtain CBF values. ASL parameters- FoV- 250 mm, matrix- 64 × 64, TR-2500, TE-12.0, NEX- 1, slice thickness-8 mm, Inversion time-1800ms.	CBF in the cortical plate of the right and left frontal lobe	The CBF in the cortical plate of the right (39.1 ± 5.6 vs. 45.8 ± 3.2 ml/100 g/min) and left (39.2 ± 6.2 vs. 45.2 ± 3.6 ml/100 g/min) frontal lobe was significantly (*P* < 0.001) lower in patients with arterial hypertension compared to the controls. 51.5% of hypertensive patients had white matter hyperintensities.	The study did not assess CBF in other lobes of the brain.

### Participant characteristics

The total number of participants studied is 2296, with an average of 328. The lowest number of participants in a study is 37, and the study with the highest participant number is 902. Both male and female participants were included in the study, and participants ranged in age from 40 to 90 years. The characteristics of the participants included in the study are shown in Table 1.

### Hypertension and its influence on cerebral perfusion

All studies had a shared focus on exploring the influence of high blood pressure on CBF, and across all the studies, there was a consistent relationship between elevated blood pressure and reduced CBF. Reduced brain circulation leads to a decrease in brain structural integrity [28]. This can lead to consequences such as infarction, Dementia, cognitive decline, and Alzheimer's disease. The studies that have explored the total CBF and its association with hypertension are those by Alosco *et al.* [28] and Li *et al.* [29]. The study by Alosco *et al.* [28] has also assessed brain lobar perfusion and demonstrated decreased perfusion in the lobes of the brain, especially the frontal (hypertension:45.65 ± 7.62, nonhypertension:46.80 ± 6.57) and parietal lobe (hypertension:48.27 ± 7.62, nonhypertension:50.83 ± 7.34) as a consequence of elevated blood pressure. Global perfusion provides overall information about cerebral perfusion, but studying the brain region-wise CBF based on hypertension is vital. The hippocampus is a memory centre of the brain and plays a significant role in cognition. Hippocampal perfusion decreases with elevated blood pressure, as shown in the study conducted by Glodzik *et al.* [19], Dai *et al.* [30], and Li *et al.* [29]. The CBF values in the hippocampus, as observed by Glodzik *et al.*, were (hypertension:63.6 ± 0.6, nonhypertension:64.9 ± 0.8), whereas in the study by Dai *et al.*, they were (hypertension:30.0 ± 11.9, nonhypertension:51.8 ± 12.0). A decrease in hippocampus CBF can be a trigger for neurological disorders such as Alzheimer's disease. The study conducted by Dai *et al.* [30] has demonstrated decreased rCBF in the subcortical, limbic, and paralimbic structures such as the putamen, globus pallidus, cingulate gyrus, hippocampus, and temporal cortex. The decreased perfusion in these structures could be a marker for cognitive decline and neurological disorders in adults with hypertension.

### Hypertension treatment and its effect on cerebral blood flow

High blood pressure is often treated using antihypertensives, and this could help eliminate the consequences of hypertension, such as cardiovascular and cerebrovascular diseases. The study performed by Tryambake *et al.* [31] showed that intensive treatment for hypertension can improve the CBF significantly in the grey matter in older adults with hypertension. Similarly, the study by Christie *et al.* [32] demonstrated that medication-controlled high blood pressure improved CBF compared to uncontrolled hypertension patients. Thus, CBF measured from ASL-MRI could be used as a noninvasive technique for monitoring the effectiveness of hypertension treatment and as a marker of cerebrovascular disease.

### Quality assessment of the study

The quality assessment of the seven studies was performed using the JBI critical appraisal tool for cross-sectional studies, and the results are shown in Table 2. All the studies were of high quality, with a low risk of bias in the participant selection, measurement validity, and outcome assessment domains. There was a moderate risk of bias due to the domain of confounding factors, whereas all other domains had a low risk of bias.

**TABLE 2:** Quality assessment of the studies

Study	Q1: Criteria for Inclusion Clearly Defined	Q2: Description of Subjects and Setting	Q3: Valid and Reliable Exposure Measurement	Q4: Standard Outcome Measurement	Q5: Confounding Factors Identified	Q6: Strategies to Deal with Confounders	Q7: Valid and Reliable Outcome Measurement	Q8: Appropriate Statistical Analysis	Overall Risk of Bias
Glodzik *et al.* (2019)	Yes	Yes	Yes	Yes	Yes	Yes	Yes	Yes	Low
Alosco *et al.* (2014)	Yes	Yes	Yes	Yes	Yes	Yes	Yes	Yes	Low
Dai *et al.* (2008)	Yes	Yes	Yes	Yes	Yes	No	Yes	Yes	Low to Moderate
Li *et al.* (2023)	Yes	Yes	Yes	Yes	Yes	Yes	Yes	Yes	Low
Christie *et al.* (2022)	Yes	Yes	Yes	Yes	Yes	Yes	Yes	Yes	Low
Tryambake *et al.* (2013)	Yes	Yes	Yes	Yes	Yes	No	Yes	Yes	Low to Moderate
T.M. *et al.* (2018)	Yes	Yes	Yes	Yes	Yes	No	Yes	Yes	Low to Moderate

### Meta-analysis

**FIGURE 2:**
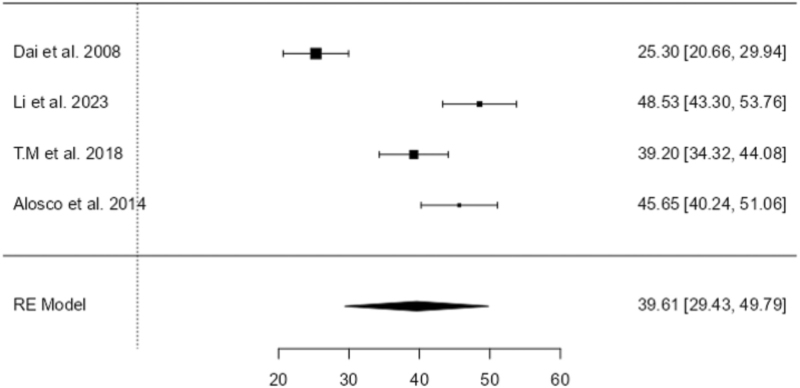
Forest plot of the frontal lobe in the hypertensive group. This plot shows the effect sizes and confidence intervals of studies assessing frontal lobe changes in individuals with hypertension.

**FIGURE 3:**
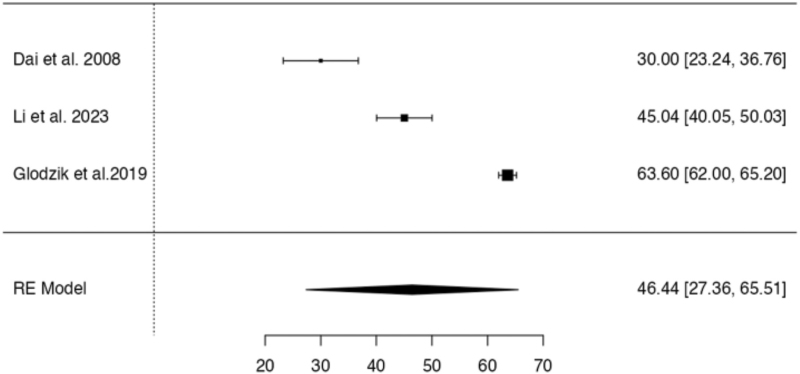
Forest Plot of the hippocampus in the hypertensive group. This plot shows the effect sizes and confidence intervals for hippocampal region among hypertensive participants.

A meta-analysis was conducted to examine the effect of hypertension on CBF in the frontal lobe and hippocampus. A random-effects model was applied for the hypertensive group due to substantial between-study heterogeneity. Four studies assessing CBF in the frontal lobe showed a statistically significant reduction of CBF among individuals with hypertension (estimate = 39.6, SE = 5.19, *Z* = 7.63, *P* < 0.001), with a 95% confidence interval (CI) of 29.43 to 49.79. Heterogeneity was high (*I*^2^ = 93.9%, *Q* = 51.99, *P* < 0.001), indicating considerable variability in study effect sizes. Similarly, for the hippocampus, three studies showed significantly reduced CBF in hypertensive individuals (estimate = 46.4, SE = 9.73, *Z* = 4.77, *P* < 0.001; 95% CI: 27.36–65.51), with substantial heterogeneity (*I*^2^ = 98.34%, *Q* = 129.40, *P* < 0.001). Figures 2 and 3 show the forest plots of the frontal lobe and hippocampus region in the hypertensive group.

**FIGURE 4:**
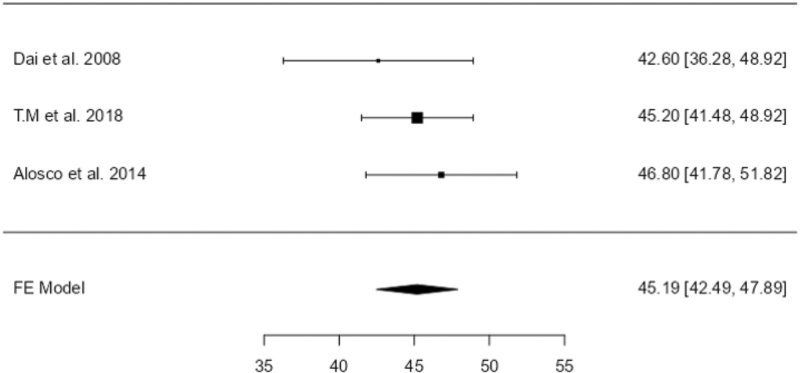
Forest Plot of the frontal lobe in the nonhypertensive group. This plot shows the effect sizes and confidence intervals of studies evaluating frontal lobe changes in nonhypertensive individuals.

A fixed-effects model was used for the nonhypertensive group, as no significant heterogeneity was observed (*I*^2^ = 0%, *Q* = 1.04, *P* = 0.595). The pooled estimate from three studies showed a significantly higher and consistent CBF in the frontal lobe (estimate = 45.2, SE = 1.38, *Z* = 32.8, *P* < 0.001), with a narrow 95% CI of 42.49–47.89. The forest plot for this analysis is shown in Fig. 4.

Meta-analyses for other brain regions and the hippocampus in the nonhypertensive group were not conducted due to insufficient studies and data. However, the results indicate a clear and statistically significant association between hypertension and reduced CBF in both the frontal lobe and hippocampus, while CBF in nonhypertensive individuals remains stable and consistently higher in the frontal lobe.

## DISCUSSION

This systematic review aimed to consolidate current research on the impact of hypertension on CBF, as measured by ASL-MRI. The findings indicate a consistent association between elevated blood pressure and reduced CBF, particularly in brain regions critical for cognitive function, such as the hippocampus, subcortical regions, and temporal lobes. The reduction in CBF [31,32] observed in these vulnerable regions highlight significant implications for neurovascular and cognitive health. These regions are also vulnerable to neurodegenerative diseases such as Alzheimer's disease, suggesting that hypertension may accelerate cognitive decline [19,29]. This is supported by studies conducted by Glodzik *et al.* and Dai *et al.*, which observed decreased CBF in limbic and paralimbic regions.

While ASL-MRI offers a promising noninvasive approach for quantifying CBF, its application in hypertension research and clinical care requires careful consideration. Although studies such as those by Tryambake *et al.* and Christie *et al.* have demonstrated improved cerebral perfusion in hypertensive adults with well controlled blood pressure, the overall differences in CBF between hypertensive and normotensive groups tend to be small and often fall within the range of normal inter-individual variability. This is partly due to the known sensitivity of ASL-MRI to external and physiological factors such as time of day, caffeine intake, scanner settings, etc., which contributes to substantial within-group variability. As such, while ASL-MRI may hold potential for monitoring treatment-related trends at the group level, its application in individual-level assessment remains uncertain. The utility of ASL-MRI in clinical settings for individual-level assessment requires Significant standardization of imaging protocols, subject preparation, and analysis methods. Future research should also focus on establishing normative reference values and thresholds to improve its diagnostic and prognostic relevance in hypertension.

All the studies included in this review were conducted in high-income and upper-middle-income countries, leaving a gap in understanding the impact of hypertension on CBF in low-income countries. Since hypertension is prevalent in low and middle-income countries, as the WHO (2015) reported [6]. Future research should also include low-income and lower-middle-income countries to better represent global populations and ensure that the findings are generalizable across different income groups.

Quantitative assessment of CBF using ASL-MRI potentially allows for the use of threshold or reference values, thus aiding in the differentiation between healthy and diseased conditions. However, several physiological and technical factors can affect the accuracy and reproducibility of CBF measurements. These include age-related changes in perfusion, the influence of recent caffeine consumption, sleep deprivation, and baseline metabolic activity. Age is a critical determinant, as younger individuals typically exhibit higher CBF due to elevated metabolic demands, while perfusion decreases progressively with aging [33,34]. Caffeine intake can significantly reduce CBF by inducing cerebral vasoconstriction, and therefore, caffeine abstinence is recommended before imaging to avoid artificially low perfusion values [35,36]. Sleep deprivation and circadian variations also influence CBF, particularly in brain regions associated with cognitive and emotional processing. This highlights the importance of documenting and adjusting for these factors during image acquisition [37]. From a technical standpoint, the partial volume effect, caused by the large voxel size used in ASL-MRI, can lead to inaccurate quantification, especially in atrophic or heterogeneous tissues. This can be corrected using the postprocessing technique called partial volume correction, which improves the accuracy of CBF estimates [38]. The ongoing initiatives, such as the Open Science Initiative for Perfusion Imaging (OSIPI) and emerging frameworks like STEPS for standardized reporting in perfusion MRI, aim to support standardization and reproducibility in ASL-MRI research by addressing methodological variability, promoting best practices, and facilitating the development of image processing pipelines that can serve as practical guides for novice ASL users [39,40].

Cerebrovascular reactivity is vital in preserving cerebral autoregulation and ensuring consistent CBF across various arterial pressures. However, the intricate relationship between the brain tissue and the cerebrovascular system changes as we age, adversely affecting brain health and cognitive function [41,42]. Most of the studies included in this review have studied the effects of hypertension and its effect on CBF among the elderly population, leaving a gap in understanding the influence of high BP in the young and middle-aged population. Future studies should focus on young and middle-aged populations to study the effects of hypertension on cerebral perfusion without the influence of aging.

It is widely recognized that hypertension varies between males and females as they age [42]. Studies have shown that the prevalence of hypertension in males up to the age of 50 years is higher; after that, females have higher rates [43]. Despite these known differences, none of the studies included in this review assessed CBF using ASL-MRI in relation to gender among hypertensive individuals. Moreover, most included studies had a slightly higher proportion of female participants, yet none performed gender-specific analysis of CBF. This highlights an essential gap in the literature and underscores the need for future research to explore gender-based variations in CBF among individuals with hypertension.

The duration and onset of hypertension may influence cerebral changes, including reductions in CBF. However, this aspect was not adequately addressed in most of the studies included in our review. Only one study by T.M. *et al.* 2018 [33] reported the duration of hypertension, which ranged between 2 and 3 years, while the remaining studies did not specify the duration or onset. As such, we could not draw conclusions regarding the relationship between the duration of hypertension and CBF. This represents a significant limitation in the existing literature and our review. Future research should aim to systematically report the duration and onset of hypertension, as this could be an essential factor influencing the degree of CBF reduction observed.

## CONCLUSION

This systematic review highlights a consistent association between hypertension and reduced CBF, particularly in regions critical for cognition, such as the hippocampus. Studies using ASL-MRI demonstrate that elevated blood pressure leads to significant decreases in CBF, which can contribute to cognitive decline and neurodegenerative diseases like Alzheimer's. The findings also emphasize the utility of ASL-MRI as a noninvasive tool in assessing CBF in hypertensive patients and monitoring the efficacy of antihypertensive treatments. However, future studies should address the research gaps identified in this study to gain complete insights into the association of CBF and hypertension using ASL-MRI. This evidence is crucial for guiding future research and public health strategies.

## ACKNOWLEDGEMENTS

The authors sincerely thank Manipal Academy of Higher Education (MAHE) University for their invaluable support and resources, which were instrumental in the successful completion of this systematic review.

Funding statement: This research received no specific grant from any funding agency, commercial, or not-for-profit sectors.

### Conflicts of interest

There are no conflicts of interest.
